# Preclinical validation of a novel deep learning‐based metal artifact correction algorithm for orthopedic CT imaging

**DOI:** 10.1002/acm2.14166

**Published:** 2023-10-03

**Authors:** Rui Guo, Yixuan Zou, Shuai Zhang, Jiajia An, Guozhi Zhang, Xiangdong Du, Huan Gong, Sining Xiong, Yangfei Long, Jing Ma

**Affiliations:** ^1^ Department of Radiology Xinjiang Production & Construction Corps Hospital Urumqi China; ^2^ United Imaging Healthcare Shanghai China

**Keywords:** artificial intelligence, computed tomography, metal artifacts correction, musculoskeletal imaging, virtual monochromatic imaging

## Abstract

**Purpose:**

To validate a novel deep learning‐based metal artifact correction (MAC) algorithm for CT, namely, AI‐MAC, in preclinical setting with comparison to conventional MAC and virtual monochromatic imaging (VMI) technique.

**Materials and methods:**

An experimental phantom was designed by consecutively inserting two sets of pedicle screws (size Φ 6.5 × 30‐mm and Φ 7.5 × 40‐mm) into a vertebral specimen to simulate the clinical scenario of metal implantation. The resulting MAC, VMI, and AI‐MAC images were compared with respect to the metal‐free reference image by subjective scoring, as well as by CT attenuation, image noise, signal‐to‐noise ratio (SNR), contrast‐to‐noise ratio (CNR), and correction accuracy via adaptive segmentation of the paraspinal muscle and vertebral body.

**Results:**

The AI‐MAC and VMI images showed significantly higher subjective scores than the MAC image (all *p* < 0.05). The SNRs and CNRs on the AI‐MAC image were comparable to the reference (all *p* > 0.05), whereas those on the VMI were significantly lower (all *p* < 0.05). The paraspinal muscle segmented on the AI‐MAC image was 4.6% and 5.1% more complete to the VMI and MAC images for the Φ 6.5 × 30‐mm screws, and 5.0% and 5.1% for the Φ 7.5 × 40‐mm screws, respectively. The vertebral body segmented on the VMI was closest to the reference, with only 3.2% and 7.4% overestimation for Φ 6.5 × 30‐mm and Φ 7.5 × 40‐mm screws, respectively.

**Conclusions:**

Using metal‐free reference as the ground truth for comparison, the AI‐MAC outperforms VMI in characterizing soft tissue, while VMI is useful in skeletal depiction.

## 1 INTRODUCTION

Metallic orthopedic implants are often seen in joint and spinal surgery, where computed tomography (CT) is an important tool to both pre‐operative planning and post‐operative following up. However, CT imaging is in principle vulnerable to metal implants. The metal artifacts that appear as bright and dark streaks on CT image can severely degrade the image quality, affecting both the observation and the quantitative analysis of peri‐implant regions.^1^, ^2^ It challenges the management of broken, loose, misplaced implants, the diagnosis of implant induced infection as well as the detection of lesions within a distance.^3^ In clinical practice, the only reliable way to tackle with these issues may be to follow the patient over a long term.

Since 1980s, the problem of metal artifact has been attacked by investigations using various metal artifacts reduction (MAR) techniques.^4^, ^5^ In this regard, dual‐energy CT has a natural advantage in reducing metal artifacts, for being able to produce monoenergetic images, often referred as virtual monoenergetic imaging (VMI). It has been proven by numerous studies that 100–140 keV is the optimal range of mono‐energies for observing images involving metal implants.^6^, ^7^, ^8^, ^9^, ^10^

Software approaches has been proven a successful alternative to deal with metal artifacts, which are known as metal artifacts correction (MAC) algorithms. The classical method follows the idea of projection completion proposed by Kalendar et al.^11^ Over time, a variety of such algorithms have been developed and put in commercial use, despite the fact that the performance in case of metal objects with complex geometry is still not satisfying.^5^ Lately, deep learning technique is being introduced to the field of CT imaging, showing promise as a new concept for image reconstruction that can be particularly useful for low dose acquisitions.^12^, ^13^ This inspires a similar thinking whether deep learning can also be utilized to benefit MAC algorithms.

Currently, an artificial intelligence (AI)‐MAC (AI‐uMAC, United Imaging Healthcare, Shanghai, China) algorithm is being actively developed using deep learning, which is fundamentally different from the conventional MAC. Before test on patients, it is crucial to validate this novel algorithm first in preclinical setting, where the ground truth is provided via experimentally designed phantom for side‐by‐side evaluation. With qualitative and quantitative comparisons to the VMI and conventional MAC, this study aimed to score the performance of the AI‐MAC and determine the scenarios where it may be a superior choice in musculoskeletal CT.

## 2 MATERIALS AND METHODS

This experimental study involved the use of purchased animal specimen, which was reviewed and approved by the Institutional Review Board of the Xinjiang Production & Construction Corps Hospital.

### 2.1 Phantom design

**FIGURE 1:**
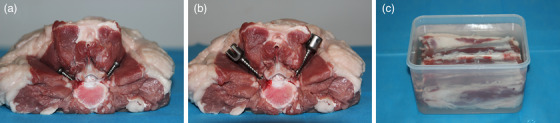
Ovine vertebra phantom. (a) The Φ 6.5 × 30‐mm screws implanted vertically into the bilateral pedicles. (b) The Φ 7.5 × 40‐mm screws implanted along the previous implantation trajectories. (c) The water base for the vertebral specimen.

As shown in Figure 1, the experimental phantom consisted of three components: a water base, an ovine vertebral specimen, and the metal object to be inserted. The vertebral specimen was obtained from a 12‐month‐old sheep on the day of slaughter, with a size of 14, 16, and 10 cm in three dimensions. To retain the realism in anatomy, the vertebra, muscle, and fat of the specimen were well preserved. Two sets of pedicle screws, made of stainless steel, 6.5 × 30 mm and 7.5 × 40 mm in diameter × length, were used as the metal inserts, where the size difference may also impact the MAR technique under investigation. An orthopedic surgeon with 5 years of experience in spine implantation was invited to perform the insertion. To ensure the same insert position, the smaller set of screws were implanted first, in vertical direction to the bilateral pedicles of the vertebral body. After the scan, they were removed and the larger set of screws were implanted following the same implantation trajectories. The water base was filled with normal saline, with a size comparable to that of an adult human abdomen.

### 2.2 Image acquisition

The scan was performed with a 320‐row CT scanner (uCT 960+, United Imaging Healthcare, Shanghai, China). Prior to the insertion, the phantom was scanned using the routine single‐energy protocol in order to establish a metal‐free reference. Then, the phantom with inserts was scanned twice, one in single‐energy and one in dual‐energy mode. The parameters for the single‐energy scan were: 120 kVp tube voltage, 100 mAs reference tube current, 1.0 pitch, and 0.8 s rotation time. The settings for the dual‐energy scan were: 80/140 kVp tube voltage, 600/80 mAs reference tube current, and 0.8 s rotation time.

The metal‐free reference image was reconstructed using a hybrid iterative reconstruction (HIR) algorithm (Karl 3D, United Imaging Healthcare, Shanghai, China). The single‐energy CT data obtained on the phantom with metal inserts were reconstructed using HIR combined with the conventional MAC (uMAC, United Imaging Healthcare, Shanghai, China) and with the AI‐MAC algorithm. The conventional MAC is based on the methodology as reported by Esther et al.^14^ The acquired dual‐energy data were reconstructed with HIR and processed on a dedicated post‐processing workstation (uWS‐CT, United Imaging Healthcare, Shanghai, China) in order to generate the VMI images, among which the optimal kilo‐electron‐volt (keV) for the best image quality with the least visible metal artifact and most confident image interpretability was determined by the observing radiologist.

All images were reconstructed at a 1.0‐mm slice thickness and 1.0‐mm increment with a medium‐smooth kernel.

### 2.3 Artificial intelligence metal artifact correction algorithm

**FIGURE 2:**
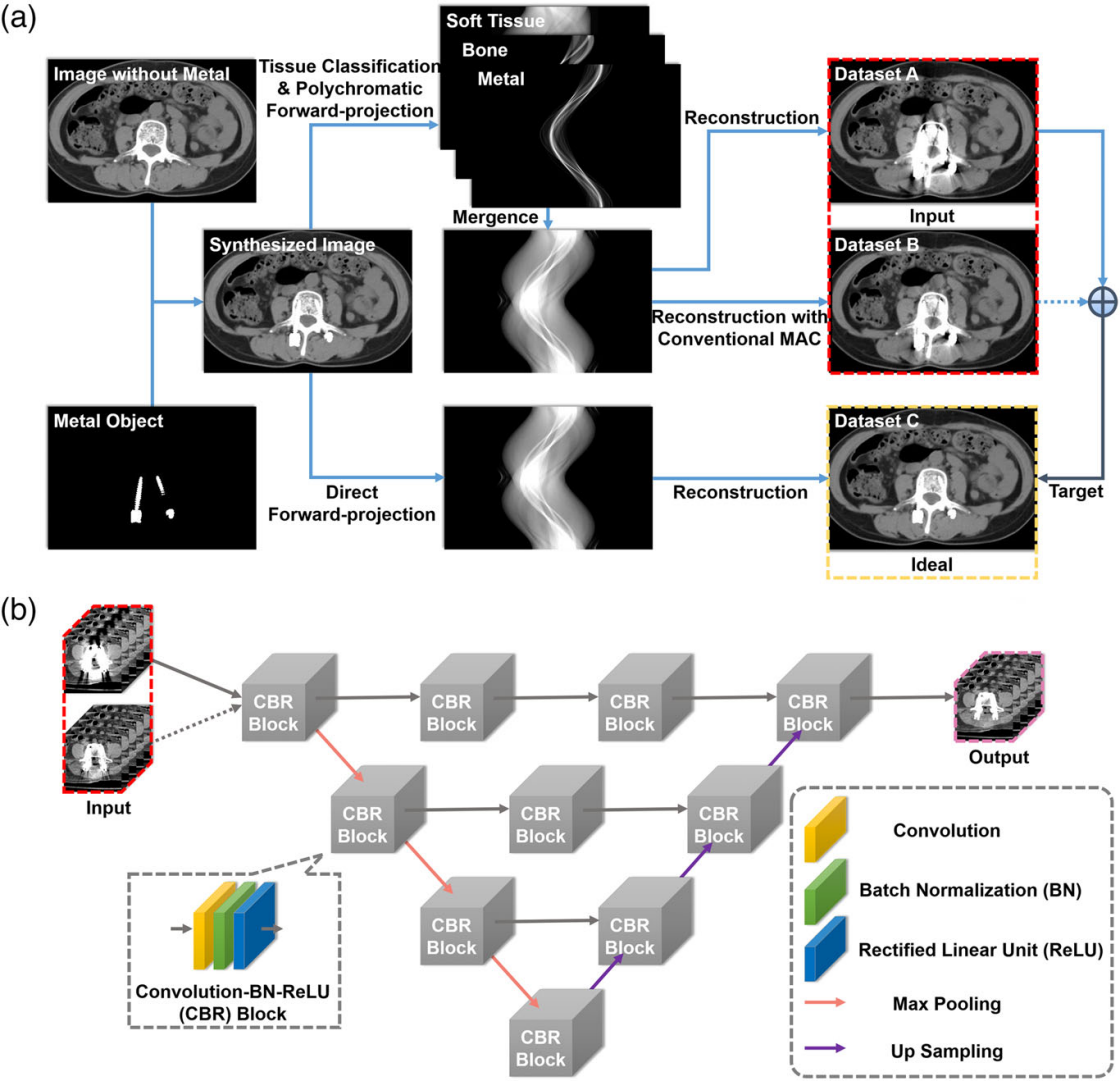
Working principle of the artificial intelligence metal artifacts correction (AI‐MAC) algorithm. (a) Preparation of the datasets. (b) Structure of the convolutional neural network.

The development of AI‐MAC algorithm can be broken into a dataset preparation and a deep learning part. Figure 2(a) shows the schematic for dataset preparation. A regular CT image without presence of metal is fused in image domain with a predefined model of metal insert, that is, its geometric shape and theoretical CT attenuation, in order to synthesize a mixed but artifact‐free image. On the one hand, this synthesized image is roughly classified into three materials, that is, bone, metal, and soft tissue, and forward projected using a polychromatic spectrum, physically simulating the generation of metal artifacts, which is next merged for reconstruction without and with the conventional MAC, producing two datasets with the original and the suppressed artifact appearance, respectively. On the other hand, the synthesized image is mathematically directly forward projected and then reconstructed, regularizing the border of insertion and producing a dataset C that would be the ideal state for correction and is defined as the target for subsequent training. Figure 2(b) shows the convolutional neural network, which is based on a modification of the widely used U‐net.^15^ It takes datasets A and B as the input and learns to minimize the metal artifacts as presented on A with respect to dataset C. Of note is that dataset B is an optional input which is only used to accelerate the convergence of deep‐learning model.

In training the deep‐learning model, an increasing collection of data has been utilized. At the time of the current investigation, regular metal‐free CT images were only selected from the chest, abdomen, and pelvis examinations, including thousands of image sets acquired from four different scanner models of the same manufacturer (United Imaging Healthcare, Shanghai, China). As for the metal implants, only screws and hip prostheses have been taken into account at this stage. While the type of examination and metal implants certainly determines the application scenarios that remain expandable subject to the growing amount of training data, the current deep‐learning model has already included various settings of the acquisition geometry and a rather broad set of X‐ray spectrum (up to 150 kVp) such that physical characteristics in metal artifact dissemination could be sufficiently picked up for correction.

### 2.4 Qualitative analysis

Six continuous slices centered on the screws were extracted from each image, anonymized, and presented in random order for qualitative analysis. Two radiologists, with 4 and 7 years of experience in musculoskeletal radiology, were asked to score the image quality per slice in subjective fashion. All images were initially presented in the soft‐tissue window (width/level = 300/45 HU), which was allowed to be changed by the radiologists at their discretion. Subjective scoring was performed in terms of the extent of metal artifacts, visualization of paraspinal muscles, completeness of vertebrae, depiction of pedicle screws, and overall image interpretability using a 5‐point Likert scale. The detailed grading criteria are shown in Table 1.

**TABLE 1:** Scoring criteria of image quality.

Image quality metric	Scoring criteria
Metal artifacts extent	Score 1, severe artifacts, occupying more than 80% of the field of view; Score 2, substantial artifacts, occupying 60%–80% of the field of view; Score 3, moderate artifacts, occupying 40%–60% of the field of view; Score 4, minor artifacts, occupying 20%–40% of the field of view; Score 5, negligible artifacts, occupying less than 20% of the field of view.
Paraspinal muscles visualization	Score 1, poor visualization, severely incomplete contours; Score 2, inadequate visualization, significant deviation from the reference image; Score 3, moderate visualization, partial deviation from the reference image; Score 4, good visualization, minor deviation from the reference image; Score 5, excellent visualization, consistent with the reference image.
Vertebrae completeness	Score 1, poor completeness, severely incomplete and asymmetrical cortical bone; Score 2, inadequate completeness, significant discontinuous cortical bone; Score 3, moderate completeness, partial discontinuous cortical bone; Score 4, good completeness, minor discontinuous cortical bone; Score 5, excellent completeness, complete cortical bone consistent with the reference image.
Pedicle screws depiction	Score 1, poor depiction, completely unrecognizable and more like cones; Score 2, inadequate depiction, rough screws shape; Score 3, moderate depiction, partly blurred screw threads, screw heads and screw slots; Score 4, good depiction, evident screw threads, screw heads and screw slots; Score 5, excellent depiction, sharply delineated screw threads, screw heads and screw slots.
Overall image interpretability	Score 1, poor interpretability, completely impossible for diagnosis; Score 2, inadequate interpretability, limited diagnostic value; Score 3, moderate interpretability, acceptable for diagnosis; Score 4, good interpretability, sufficient diagnostic value; Score 5, excellent interpretability, completely available for diagnosis.

### 2.5 Quantitative analysis

The difference between the reference image and the other images was evaluated in terms of CT attenuation, image noise, signal‐to‐noise ratio (SNR), and contrast‐to‐noise ratio (CNR). Image noise was defined by the standard deviation (SD) within the region of interest (ROI). A radiologist with 5 years of diagnostic experience was asked to delineate two ROIs relative to the region of screw implantation: one on the trabecular bone and one on the paraspinal muscle, both approximately 20 mm^2^ in size. Each ROI was measured six times across the axial image slices where the screws were most visible. The SNR was calculated as follow:

(1) SNR=μ/SD

The CNR between the bone and the muscle was calculated as follow:

(2) $$CNR\;=\;\left(\mu_{\mathrm{bone}}-\mu_{\mathrm{muscle}}\right)/\sqrt{{SD}_{\mathrm{bone}}^{2}+{SD}_{\mathrm{muscle}}^{2}}$$

where μ and SD represent the mean and the standard deviation of the CT attenuation within the ROI, in Hounsfield units (HU).

### 2.6 Correction accuracy analysis

The accuracy of different MAR techniques was evaluated for their ability to preserve the surrounding muscular and skeletal regions of metal implants. This was quantified by measuring the segmented volume of the paraspinal muscle and vertebral body using a semi‐automatic adaptive threshold segmentation program on the same post‐processing workstation mentioned above. The ranges of CT attenuation for muscle and bone were determined to be 20−100 HU and 300−1000 HU, respectively, based on the reference image. Segmentation was performed by the radiologist using 3D region growing with seed points placed in regions without noticeable metal artifacts on the paraspinal muscle and the vertebral body. The volume of interest encompassed the entire phantom, including the water base. The resulting number of voxels falling within the defined threshold range provided a quantitative measure of paraspinal muscle and vertebral body volume in the images. Comparisons were conducted between the reference image and the other images using the same segmentation. These results have important implications for accuracy of the MAR techniques in musculoskeletal quantification.

### 2.7 Statistical analysis

A commercially available software (SPSS Version 25, IBM. Armonk, New York, USA) was used for statistical analyses. Unless otherwise specified, all data are presented as mean average ± standard deviation. The qualitative scores were compared using the Wilcoxon signed‐ranks test. The Cohen's kappa test was used to assess the inter‐reader agreement (excellent, κ = 0.81–1.00; good, κ = 0.61–0.80; moderate, κ = 0.41–0.60; fair, κ = 0.21–0.40; poor, κ = 0.00–0.20). The CT attenuation, SD, SNR, and CNR were compared using the Mann–Whitney *U* test. A *p* < 0.05 was considered statistically significant.

## 3 RESULTS

### 3.1 Qualitative scores

**FIGURE 3:**
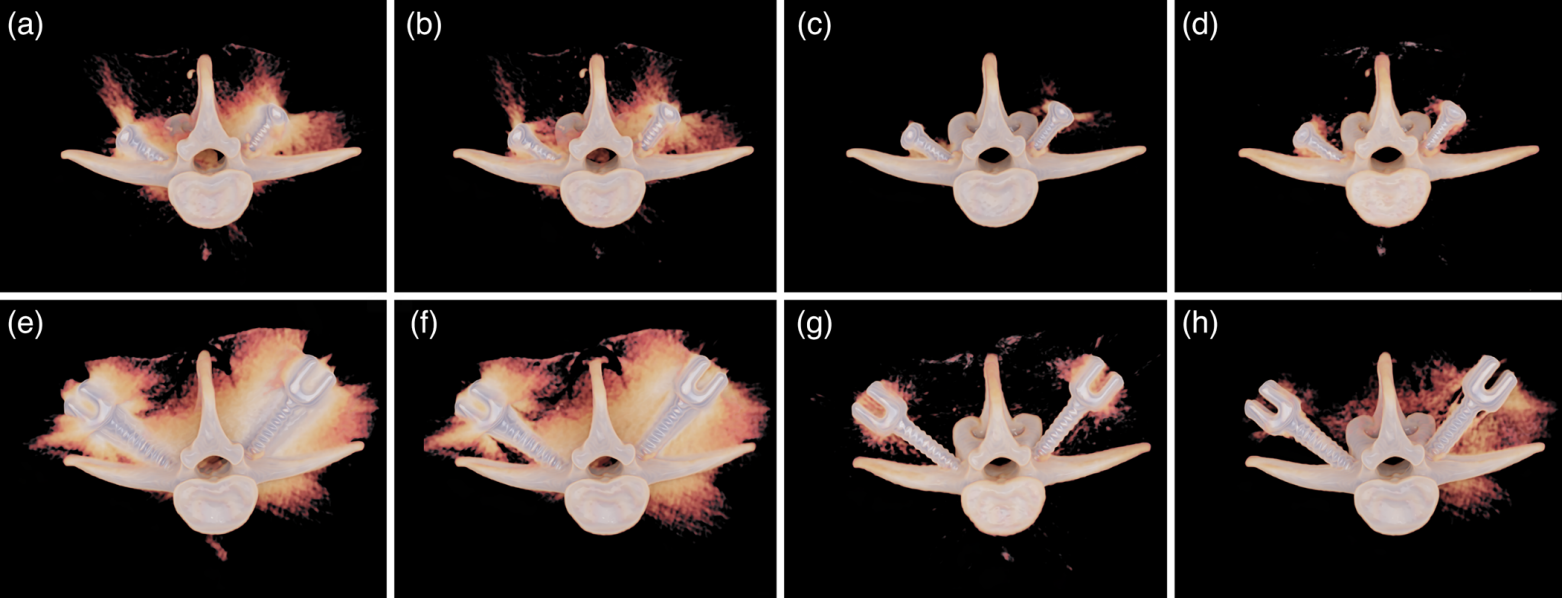
3D visual demonstration (Hyper‐Realistic Rendering, United Imaging Healthcare, Shanghai, China) of the metal artifacts extent on the images of the phantom with the Φ 6.5 × 30‐mm (a–d) and Φ 7.5 × 40‐mm (e–h) screws. (a) and (e) uncorrected image. (b) and (f) MAC image. (c) and (g) VMI image. (d) and (h) AI‐MAC image. AI‐MAC, artificial intelligence metal artifacts correction; MAC, metal artifact correction; VMI, virtual monochromatic imaging.

The 120–140 keV VMI images were rated by radiologist as the best virtual monoenergetic range for viewing image involving metal implants, where the 140 keV VMI was selected for all subsequent scoring and analysis. Table 2 shows the qualitative results acquired on uncorrected, MAC, VMI, and AI‐MAC images. The uncorrected and MAC images were scored significantly lower than VMI and AI‐MAC image in all metrics (all *p* < 0.05). In the subjective scoring of the image quality of the phantom with the Φ 6.5 × 30‐mm screws, the AI‐MAC image showed significant less artifact extent than VMI image (*p* = 0.046). But, with the Φ 7.5 × 40‐mm screws, no significant difference relative to metal artifacts was found between VMI and AI‐MAC images (*p* = 0.564). Figure 3 demonstrates the extent of the metal artifacts on different images, represented in 3D rendering.

**TABLE 2:** Resulting qualitative scores of the uncorrected, MAC, VMI, and AI‐MAC images with the phantom using the Φ 6.5 × 30‐mm and Φ 7.5 × 40‐mm screws.

	Φ 6.5 × 30‐mm screws				Φ 7.5 × 40‐mm screws			
Image quality metrics	Uncorrected	MAC	VMI	AI‐MAC	Uncorrected	MAC	VMI	AI‐MAC
Metal artifacts extent	1.25 ± 0.45	1.17 ± 0.39	4.25 ± 0.45	4.58 ± 0.51	1.00 ± 0.00	1.08 ± 0.29	4.17 ± 0.39	4.08 ± 0.29
Paraspinal muscles visualization	1.25 ± 0.45	2.25 ± 0.45	4.67 ± 0.49	4.75 ± 0.45	1.00 ± 0.00	1.08 ± 0.29	3.50 ± 0.52	3.83 ± 0.72
Vertebrae completeness	2.75 ± 0.45	3.42 ± 0.51	4.42 ± 0.51	4.50 ± 0.52	2.33 ± 0.49	3.08 ± 0.29	4.17 ± 0.39	4.08 ± 0.29
Pedicle screws depiction	2.92 ± 0.67	3.58 ± 0.51	4.83 ± 0.39	4.67 ± 0.49	1.50 ± 0.52	2.67 ± 0.49	3.92 ± 0.29	4.33 ± 0.49
Overall image interpretability	1.25 ± 0.45	2.42 ± 0.51	4.25 ± 0.45	4.75 ± 0.45	1.08 ± 0.29	2.33 ± 0.49	3.67 ± 0.49	3.83 ± 0.58

Abbreviations: AI‐MAC, artificial intelligence metal artifacts correction; MAC, metal artifact correction; VMI, virtual monochromatic imaging.

With both the Φ 6.5 × 30‐mm and Φ 7.5 × 40‐mm screws, the scoring of paraspinal muscles visualization and vertebrae completeness showed no significant difference between VMI and AI‐MAC images (all *p* > 0.05). In the case of the depiction of the Φ 6.5 × 30‐mm screws, VMI image score the best among other images. But, VMI and AI‐MAC images showed no statistically significant difference in terms of screws depiction (*p* = 0.157). With regard to the Φ 7.5 × 40‐mm screws, AI‐MAC image was superior to VMI image in terms of exact depiction of screws (*p* = 0.025). Regarding the overall image interpretability, the AI‐MAC showed better performance than VMI image with the Φ 6.5 × 30‐mm screws (*p* = 0.014). However, in the CT imaging of the phantom with the Φ 7.5 × 40‐mm screws, no statistically significant difference was seen for VMI and AI‐MAC images (*p* = 0.480). Additionally, there was good to excellent agreement between the two radiologists, with κ value lying between 0.75 and 0.82.

### 3.2 Quantitative results

Table 3 presents the CT attenuation and image noise measurements obtained from two regions of interest for all images. The uncorrected image demonstrated the most severe metal artifact‐induced degradation in CT imaging of the phantom with the Φ 6.5 × 30‐mm screws, as indicated by both CT attenuation and image noise that were significantly higher than those of the reference image (all *p* < 0.001). However, the AI‐MAC effectively corrected metal artifacts. On the AI‐MAC image with the Φ 6.5 × 30‐mm screws, both CT attenuation and image noise showed no significant difference compared to those on the reference images (all *p* > 0.05).

**TABLE 3:** Mean CT attenuation and noise of the reference, uncorrected, MAC, VMI, and AI‐MAC images with the phantom using the Φ 6.5 × 30‐mm and Φ 7.5 × 40‐mm screws.

		Φ 6.5 × 30‐mm screws				Φ 7.5 × 40‐mm screws			
	Reference	Uncorrected	MAC	VMI	AI‐MAC	Uncorrected	MAC	VMI	AI‐MAC
Bone									
CT attenuation (HU)	363.98 ± 33.58	579.90 ± 56.43	440.80 ± 55.10	230.46 ± 32.25	389.79 ± 64.58	690.53 ± 91.72	526.79 ± 114.77	263.97 ± 28.88	404.83 ± 37.40
Noise (HU)	52.89 ± 2.94	74.46 ± 14.86	56.32 ± 7.36	53.56 ± 8.66	55.31 ± 5.60	82.43 ± 10.42	62.60 ± 9.58	56.43 ± 5.92	59.18 ± 4.59
Muscle									
CT attenuation (HU)	61.04 ± 4.80	102.91 ± 20.22	86.78 ± 19.84	64.50 ± 6.70	64.69 ± 4.81	128.83 ± 13.92	103.32 ± 13.69	60.28 ± 10.86	65.35 ± 8.46
Noise (HU)	11.00 ± 1.04	28.93 ± 1.51	18.78 ± 2.78	16.73 ± 4.35	12.04 ± 1.49	31.90 ± 9.02	18.77 ± 3.61	25.51 ± 1.79	13.03 ± 1.70

Abbreviations: AI‐MAC, artificial intelligence metal artifacts correction; MAC, metal artifact correction; VMI, virtual monochromatic imaging.

In CT imaging of the phantom with the Φ 7.5 × 40‐mm screws, the ability of conventional MAC algorithm to suppress metal artifacts was relatively limited, leading to significantly higher CT attenuation and image noise in bone and muscle compared to those on the reference image (all *p* < 0.001). However, on both VMI and AI‐MAC images, no significant difference was observed in the CT attenuation of muscle compared to the reference image (*p* = 0.178 and 0.799, respectively). Additionally, the VMI image exhibited no significant difference in image noise of bone compared to the reference image (*p* = 0.520).

**FIGURE 4:**
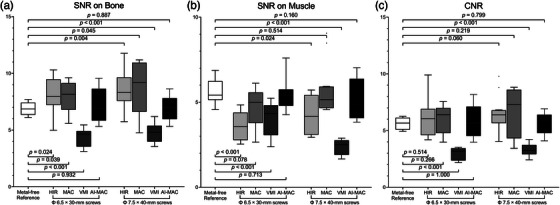
Comparison of the SNRs on bone (a), on muscle (b), and the CNRs (c) between the uncorrected, MAC, VMI, and AI‐MAC images with respect to the reference image. AI‐MAC, artificial intelligence metal artifacts correction; CNR, contrast‐to‐noise ratio; MAC, metal artifact correction; SNR, signal‐to‐noise ratio; VMI, virtual monochromatic imaging.

Figure 4 shows the differences of SNRs and CNRs between the reference image and other images. The AI‐MAC image exhibited no significant differences in SNRs and CNRs compared to those on the reference image (all *p* > 0.05). The SNRs of muscle on the MAC image was not significantly different compared to the reference image (both *p* > 0.05). However, we assumed that these results were occurring by coincidence because of the substantially increased CT attenuation and image noise of muscle on the MAC images. Furthermore, the VMI image exhibited significantly lower SNRs of bone and muscle compared to the reference image (all *p* < 0.001), and significantly lower CNRs than the reference image (all *p* < 0.001). It may be attributed to the increased proportion of high‐energy data in the dual‐energy extrapolation, which in turn results in decreased difference of CT attenuation and impaired image contrast.

### 3.3 Correction accuracy

**FIGURE 5:**
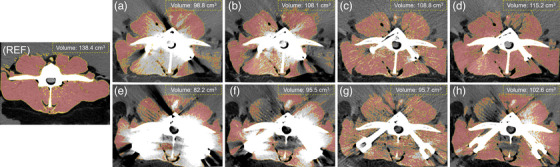
Results of adaptive muscle segmentation on the images of the phantom with the Φ 6.5 × 30‐mm (a–d) and Φ 7.5 × 40‐mm (e–h) screws (window width/level = 300/45 HU). REF, reference image. (a) and (e) uncorrected image. (b) and (f) MAC image. (c) and (g) VMI image. (d) and (h) AI‐MAC image. The segmented muscles are marked in red and outlined in yellow. AI‐MAC, artificial intelligence metal artifacts correction; MAC, metal artifact correction; VMI, virtual monochromatic imaging.

Figure 5 displays the resulting muscle segmentation obtained using reference and other images. The reference image revealed a segmented paraspinal muscle volume of 138.4 cm^3^. In CT imaging of the phantom with the Φ 6.5 × 30‐mm screws, the AI‐MAC image provided the segmentation result closest to the reference image at 115.2 cm^3^, followed by 108.8 and 108.1 cm^3^ for the VMI and MAC images, respectively. With the Φ 7.5 × 40‐mm screws, the AI‐MAC algorithm continued to outperform VMI technique and MAC algorithm, producing a segmentation result of 102.6 cm^3^, superior to 95.7 cm^3^ for the VMI image and 95.5 cm^3^ for the MAC image. Using the segmentation results of the reference image as a benchmark, the paraspinal muscle segmented on the AI‐MAC image was 4.6% and 5.1% more complete to the VMI and MAC images for the Φ 6.5 × 30‐mm screws, and 5.0% and 5.1% for the Φ 7.5 × 40‐mm screws, respectively.

**FIGURE 6:**
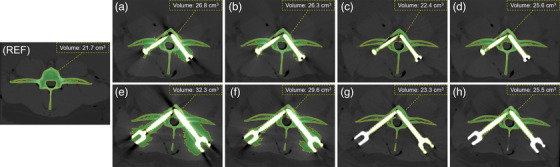
Results of adaptive vertebra segmentation on the images of the phantom with the Φ 6.5 × 30‐mm (a–d) and Φ 7.5 × 40‐mm (e–h) screws (window width/level = 1000/300 HU). REF, reference image. (a) and (e) uncorrected image. (b) and (f) MAC image. (c) and (g) VMI image. (d) and (h) AI‐MAC image. The segmented muscles are marked in green and outlined in yellow. AI‐MAC, artificial intelligence metal artifacts correction; MAC, metal artifact correction; VMI, virtual monochromatic imaging.

Figure 6 illustrates representative images of vertebral segmentation acquired with reference and other images. The reference image revealed a segmented vertebral body volume of 21.7 cm^3^. In CT imaging of the phantom with the Φ 6.5 × 30‐mm screws, the measured volumes for uncorrected, MAC, VMI, and AI‐MAC images were 26.8, 26.3, 22.4, and 25.6 cm^3^, respectively. The VMI image produced the smallest difference compared to the reference image. Similarly, with the Φ 7.5 × 40‐mm screws, the VMI image showed the closest result to the reference image for vertebra segmentation at 23.3 cm^3^, followed by 25.5 cm^3^ for the AI‐MAC image and 29.6 cm^3^ for the MAC image. For Φ 6.5 × 30‐mm and Φ 7.5 × 40‐mm screws, the segmentation overestimation of the VMI image compared to the reference image was only 3.2% and 7.4%, respectively.

## 4 DISCUSSION

In CT examination of the musculoskeletal system, handling the imaging of metal implants consists a significant portion of routine clinical practice. Metal artifacts can significantly diminish the image quality of bone and surrounding soft tissues. As a result, it might interference the CT assessment of orthopedic complications such as fistulas, spinal meningocele, and surgical site infections.^16^ In this study, we conducted a preclinical validation for a newly proposed AI‐MAC algorithm. Its ability to reduce metal artifacts and restore the accurate structure surrounding metal implants was compared versus the conventional MAC algorithm and the VMI technique.

The deep learning‐based MAC algorithm represents a significant advancement over conventional interpolation‐based MAC algorithms. A study by Arabi et al.^17^ demonstrated that a deep learning‐based MAC algorithm generated more accurate attenuation maps compared to conventional methods. In our study, we observed that the AI‐MAC algorithm surpasses conventional MAC in subjective scoring, indicating superior performance in artifact reduction. Additionally, the AI‐MAC demonstrated better artifact reduction and overall image quality the phantom with the Φ 6.5 × 30‐mm screws compared to VMI, and improved screw depiction for the Φ 7.5 × 40‐mm screws. These results highlight the potential of the AI‐MAC algorithm as a promising solution for improving the accuracy and reliability of imaging in clinical scenario.

When comparing VMI to AI‐MAC in more detail, the latter demonstrated superiority in terms of quantitative image quality. Although VMI image at high keV levels yielded a significant artifact reduction, it showed significantly reduced SNRs and CNRs compared with reference image. These findings are consistent with previous studies that have reported reduction in image contrast as a result of increased keV levels in VMI.^18^, ^19^ The decreased soft tissue contrast is a disadvantage of VMI image in musculoskeletal CT. But, with the use of VMI image, the loss of soft tissue contrast is an inevitable compromise in order to allow assessment of adjacent soft tissue in the presence of metal implants. Hackenbroch et al.^20^ reported that VMI may lead to a lack of resolution in fine structures, while Pennig et al.^21^ suggested that high keV values should be applied with caution due to the reduction in soft tissue contrast that can adversely impact diagnostic accuracy. However, the SNRs and CNRs of AI‐MAC image were not significantly different from those of the reference image. Therefore, the AI‐MAC could be a better alternative to VMI, when the diagnostic task aims for higher soft tissue contrast.

Body composition have been considered as a novel imaging biomarker, with promising performance for prediction of future adverse events.^22^, ^23^ The musculoskeletal quantification is of key importance for evaluation health condition and is associated with morbidity and mortality in a variety of diseased populations.^24^, ^25^, ^26^, ^27^ However, in the presence of metal artifacts, performing body composition analysis can be challenging. In our study, the AI‐MAC image demonstrated good performance in muscle segmentation. It indicates that AI‐MAC provides an optional method for evaluating muscular tissue in patients with metal implants. Regarding skeletal quantification, the VMI image showed the closest segmented volume to the reference image. Bone quantification reveals mineral density, and the VMI image might help assess post‐operative dynamic parameters such as bone formation and resorption. Detailed quantification allows for better diagnosis of patients with metal implants during bone remodeling. Consistent with our results, Laukamp et al.^28^ also demonstrated that the VMI was superior in depicting bone compared to conventional MAC algorithms. Based on our findings, careful selection of the appropriate metal artifact reduction technique is essential when performing body composition quantification on patients with metal implants.

This study presents certain limitations that should be acknowledged. First, the present validation remained in a preclinical setting and no test on patient was possible yet. Second, the AI‐MAC algorithm that is taken for test in this study is only one implementation of the deep learning method. Different implementations of these algorithms may lead to different outputs and warrant further investigation. Given the deep learning principle of the AI‐MAC, we would expect our findings to demonstrate the potential of deep learning technique in CT metal artifact correction. Third, the combined use of VMI and AI‐MAC algorithms might or might not show better performance than did VMI or AI‐MAC alone. Some studies have reported the combination of VMI and conventional MAC yields stronger reduction of metal artifacts.^19^, ^29^, ^30^ This is out the scope of this study, with our aim being first to determine possible advantages of one MAR technique over the other.

In summary, the AI‐MAC was found to be effective in metal artifacts reduction in CT, with superior performance to the conventional MAC algorithm. It outperformed VMI in characterizing soft tissue in regions relative to metal, while VMI retained its advantage in skeletal tissue depiction. As indicated by such preclinical findings, the AI‐MAC has the potential to be introduced into clinical application or investigation.

## AUTHOR CONTRIBUTIONS

The authors confirm contribution to the paper as follows: study conception and design: Jing Ma and Guozhi Zhang; data collection: Rui Guo, Shuai Zhang, Jiajia An, Xiangdong Du, Huan Gong, Sining Xiong, and Yangfei Long; analysis and interpretation of results: Rui Guo and Yixuan Zou; draft manuscript preparation: Rui Guo, Yixuan Zou, and Guozhi Zhang. All authors reviewed the results and approved the final version of the manuscript.

## CONFLICT OF INTEREST STATEMENT

The authors (Yixuan Zou and Guozhi Zhang) of this manuscript declare that they are employed researchers of the following company: United Imaging Healthcare. The other authors of this manuscript declare no relationship with any company whose products or services may be related to the subject matter of this article.
